# Neuroprotective Effect of Azithromycin Following Induction of Optic Nerve Crush in Wild Type and Immunodeficient Mice

**DOI:** 10.3390/ijms231911872

**Published:** 2022-10-06

**Authors:** Ofira Zloto, Alon Zahavi, Stephen Richard, Moran Friedman-Gohas, Shirel Weiss, Nitza Goldenberg-Cohen

**Affiliations:** 1 Goldschlager Eye Institute, Sheba Medical Center, Ramat Gan 5262000, Israel; 2 Sackler Faculty of Medicine, Tel Aviv University, Tel Aviv 6997801, Israel; 3 Department of Ophthalmology, Rabin Medical Center—Beilinson Hospital, Petach Tikva 4941492, Israel; 4 The Krieger Eye Research Laboratory, Felsenstein Medical Research Center, Petach Tikva 4941492, Israel; 5 The Krieger Eye Research Laboratory, Faculty of Medicine, Technion-Israel Institute of Technology, Haifa 3200003, Israel; 6 Department of Ophthalmology, Bnai Zion Medical Center, Haifa 3339419, Israel

**Keywords:** optic nerve crush, neuroprotection, azithromycin, NAION, neural injury, neural ischemia, neural inflammation

## Abstract

This study evaluated the potential neuroprotective effect of azithromycin (AZ) intraperitoneal injections in male C57Bl/6 (wild type, WT) and female NOD scid gamma (NSG) mice subjected to optic nerve crush (ONC) as a model for optic neuropathy. Histologically, reduced apoptosis and improved retinal ganglion cell (RGC) preservation were noted in the AZ-treated mice as shown by TUNEL staining—in the WT mice more than in the NSG mice. The increased microglial activation following ONC was reduced with the AZ treatment. In the molecular analysis of WT and NSG mice, similar trends were detected regarding apoptosis, as well as stress-related and inflammatory markers examining BCL2-associated X (*Bax*), heme oxygenase 1 (*Ho-1*), interleukin 1 beta (*Il1β*), superoxide dismutase 1 (*Sod1*), and nuclear factor-kappa B (*Nfkb*) levels. In the optic nerve, AZ increased the levels of expression of *Sod1* and *Nfkb* only in the WT mice and decreased them in the NSG mice. In the retinas of the WT and NSG mice, the *Bax* and *Ho-1* levels of expression decreased following the AZ treatment, while the *Sod1* and *Nfkb* expression decreased only in the WT mice, and remained stable near the baseline in the NSG mice. *Il1β* remained at the baseline in WT mice while it decreased towards the baseline in AZ-treated NSG mice. The neuroprotective effects demonstrated by the reduced RGC apoptosis in AZ-treated WT mice retinae, and in the optic nerves as stress-related and inflammatory gene expression increase. This did not occur in the immunodeficient NSG mice. AZ modulated the inflammatory reaction and microglial activation. The lack of an effect in NSG mice supports the assumption that AZ acts by immunomodulation, which is known to play a role in ONC damage. These findings have implications for the development and repurposing of drugs to preserve RGCs after acute optic neuropathies.

## 1. Introduction

Ischemic optic neuropathy (ION) refers to the infarction of any portion of the optic nerve from the chiasm to the optic nerve head. Clinically, it may be divided into anterior and posterior forms [1]. The pathogenesis of non-arteritic anterior ION (NAION) is not fully understood. NAION has been associated with acute nocturnal hypotension, sleep apnea, microvascular diseases, and Phosphodiesterase-5 inhibitor usage, but in most cases, it is unpredictable and no direct trigger is found [2,3]. Salgado et al. [4] reported an inflammatory component in the pathogenesis of NAION. In animal studies, the mechanical crush of the optic nerve posterior to the globe often serves as a model for investigating the pathogenesis of optic neuropathy and possible neuroprotective treatments [5,6].

The anti-inflammatory effect of azithromycin has been reported, especially in the mouse model of cerebral infarction and retinal ischemia, and its neuroprotective effect has also been reported [7,8,9,10,11,12]. Immunological modifications may be involved in the neuroprotective effects of azithromycin.

The neuroprotective effect of azithromycin may be effective as a treatment for NAION. Although not a model for NAION, the purpose of this study is to verify the neuroprotective effect of azithromycin using an experimental system of optic nerve crush (ONC) in mice as a model for optic nerve disease and to further verify the involvement of immunological modification.

## 2. Results

### 2.1. Histological Analysis (Day 21): RGC Survival

At 21 days after ONC, the RGC count was significantly higher in AZ-treated than untreated WT mice (30.1 ± 0.3 vs. 26.3 ± 2.8 cells/field, respectively, *p* = 0.01) and significantly higher in AZ-treated than untreated NSG mice (31.4 ± 2.3 vs. 19.4 ± 4.9 cells/field, respectively, *p* = 0.01). There were no statistically significant differences in retinal thickness between the treated and untreated WT groups (212 µ ± 20 vs. 208 µ ± 10, respectively) and NSG groups (214 µ ± 12 vs. 202 µ ± 15, respectively).

### 2.2. Immunohistochemistry Analysis (Day 21): Gliosis (GFAP)

**Figure 1:**
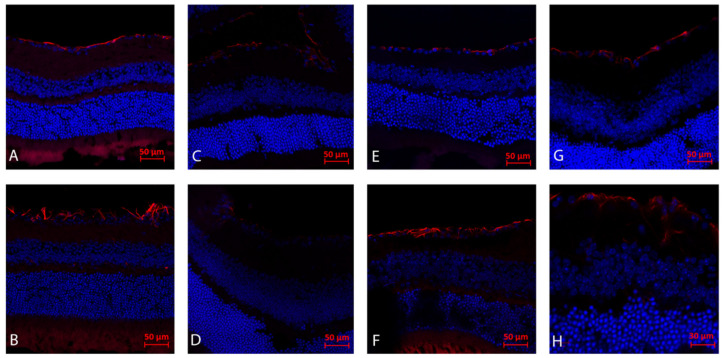
Immunohistochemistry analysis (day 21): Gliosis (GFAP). (**A**) The right eye of WT mouse following ONC and injected with AZ, demonstrating reduced gliosis and RGC preservation in comparison to right eye ONC without AZ treatment (**B**). The left eye of a WT mouse with AZ (**C**) as compared to the control left eye without AZ, (**D**) both demonstrating minimal gliosis. NSG mice following right eye ONC and systemic AZ injection demonstrates right moderate gliosis and RGC preservation, (**E**) as compared to right eye following ONC without AZ (**F**). Note that NSG mice following ONC demonstrate reduced gliosis both with (**E**) and without (**F**) AZ injection as compared to WT mice following ONC with AZ injection (**A**). NSG mice left control eye with (**G**) and without (**H**) AZ systemic injection.

The analysis following ONC in WT and NSG mice demonstrated maximal reactive gliosis and RGC loss in WT mice without the AZ treatment and a reduction in gliosis following the AZ treatment in both groups. In addition, RGC preservation can be noted in AZ-treated mice as compared to non-treated mice (Figure 1A–H).

### 2.3. TUNEL Staining (Day 1 and 3): Apoptosis

**Figure 2:**
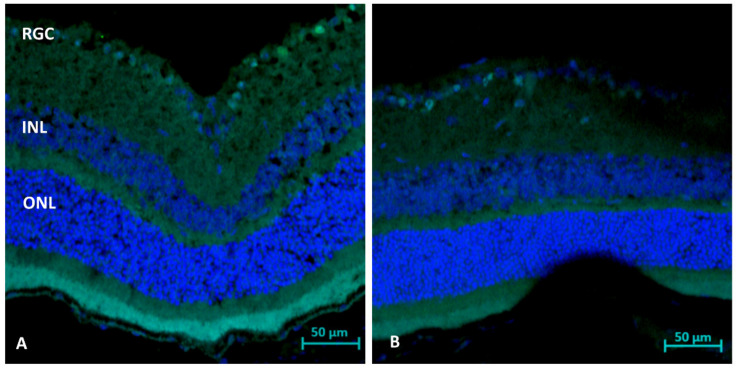
Immunohistochemistry analysis (day 3) for apoptosis (TUNEL). Note stained apoptotic RGC cells following ONC only (**A**), with reduced apoptosis following AZ treatment (**B**).

The TUNEL staining of retinal sections showed a reduction in the rate of RGC apoptosis in the AZ-treated than the untreated WT mice on day 1 (1.20 ± 0.4 vs. 2.00 ± 0.7 cells/field, respectively, *p* = 0.0081) and day 3 (4.40 ± 2.5 vs. 6.00 ± 3.7 cells/field, respectively, *p* = 0.028), respectively (Figure 2).

### 2.4. Immunohistochemistry Analysis (Day 3): Microglial Activation (Iba1)

**Figure 3:**
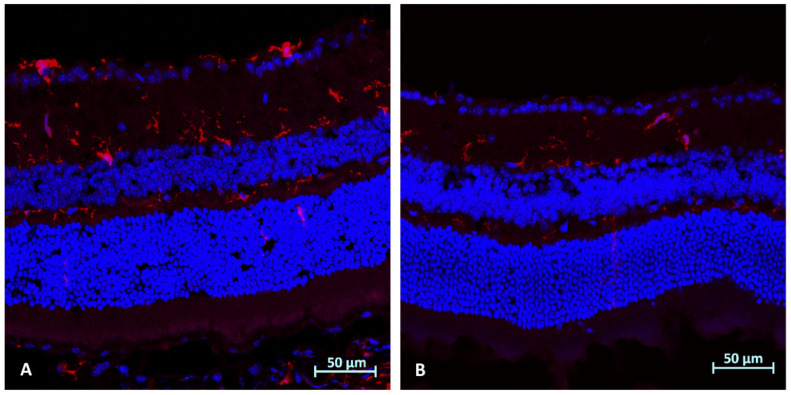
Immunohistochemistry analysis (day 3) for microglial activation (Iba1): (**A**) Right eye of WT mouse following ONC without treatment, showing increased microglial activation, as compared to AZ-treated RE ONC-induced eye (**B**), demonstrating reduced activation.

The analysis following ONC in WT mice demonstrated reduced microglial activation in AZ-treated mice (Figure 3).

### 2.5. Immunohistochemistry Analysis (Day 3): CD45

The analysis following ONC in the WT mice was nonspecific.

### 2.6. Molecular Analysis WT and NSG Mice (Day 3 after ONC): Optic Nerves

**Figure 4:**
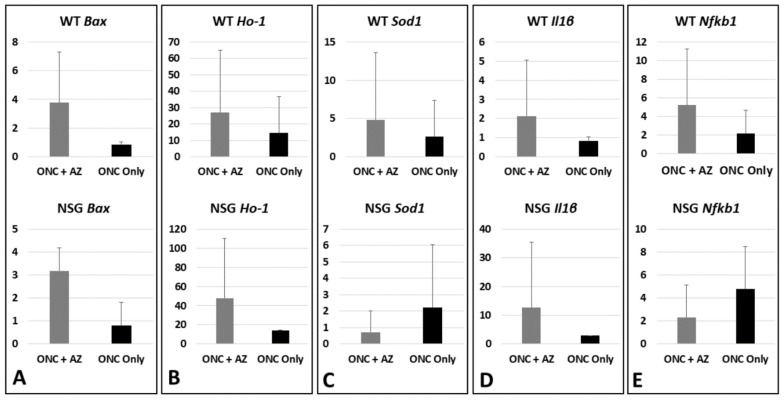
Molecular analysis of the optic nerves, day 3 after ONC. (**A**) *Bax* expression levels in WT and NSG mice remained at baseline without treatment and increased with AZ treatment. (**B**) *Ho-1* levels in WT and NSG mice increased without treatment and further increased with AZ treatment. (**C**) *Sod1* levels in WT mice increased without treatment and further increased with AZ treatment, while in NSG mice levels increased without treatment but decreased with AZ treatment. (**D**) *Il1β* levels in WT mice remained at baseline without treatment and increased with AZ treatment, while in NSG mice levels increased without treatment and further increased with AZ treatment. (**E**) *Nfkb1* levels in WT mice increased without treatment and further increased with AZ treatment, while in NSG mice levels increased without treatment with a relative decrease with AZ treatment.

*Bax*. WT mice: The *Bax* expression levels remained at the baseline without treatment (0.83 ± 0.21-fold, n = 3) and increased with the AZ treatment (3.77 ± 3.55-fold, *p* = 0.17, n = 4). NSG mice: The *Bax* expression levels remained at the baseline without treatment (0.813 ± 1.186-fold, n = 4) and increased with the AZ treatment (3.176 ± 2.262-fold, *p* = 0.087, n = 3) (Figure 4A).

*Ho-1*. WT mice: The *Ho-1* expression levels increased without treatment (14.42 ± 22.19-fold, n = 3) and further increased with the AZ treatment (27.22 ± 37.76-fold, *p* = 0.64, n = 6). NSG mice: The *Ho-1* expression levels increased without treatment (14.25 fold, n = 1) and further increased with the AZ treatment (47.73 ± 62.42-fold, n = 3) (Figure 4B).

*Sod1*. WT mice: The *Sod1* expression levels increased without treatment (2.64 ± 4.75-fold, n = 8) and further increased with the AZ treatment (4.83 ± 8.76-fold, *p* = 0.46, n = 9). NSG mice: The *Sod1* expression levels increased without treatment (2.22 ± 3.84-fold, n = 5) and decreased with the AZ treatment (0.73 ± 1.27-fold, *p* = 0.386, n = 4) (Figure 4C).

*Il1β*. WT mice: The *Il1β* expression levels remained at baseline without treatment (0.81 ± 0.23-fold, n = 4) and increased with the AZ treatment (2.12 ± 2.94-fold, *p* = 0.82, n = 7). NSG mice: The *Il1β* expression levels increased without treatment (2.94 fold, n = 1) and further increased with the AZ treatment (12.64 ± 22.81-fold, n = 4) (Figure 4D).

*Nfkb1*. WT mice: The *Nfkb1* expression levels increased without treatment (2.15 ± 2.54-fold, n = 7) and further increased with the AZ treatment (5.21 ± 6.10-fold, *p* = 0.24, n = 7). NSG mice: The *Nfkb1* expression levels increased without treatment (4.811 ± 3.676-fold, n = 3), with a relative decrease with the AZ treatment (2.304 ± 2.845-fold; *p* = 0.139, n = 4) (Figure 4E).

### 2.7. Molecular Analysis WT and NSG Mice (Day 3 after ONC): Retinas

**Figure 5:**
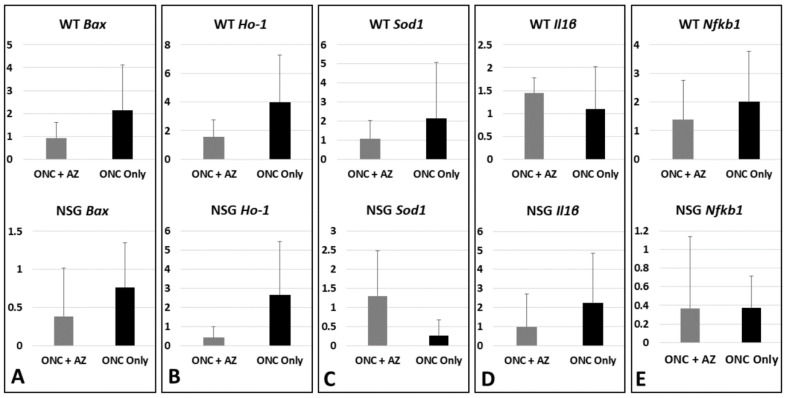
Molecular analysis of the retinas, day 3 after ONC. (**A**) *Bax* expression levels in WT mice increased without treatment and decreased with AZ treatment, while in NSG mice levels remained at baseline without treatment and decreased with AZ treatment. (**B**) *Ho-1* levels in both WT and NSG mice increased without treatment and decreased with AZ treatment. (**C**) *Sod1* levels in WT mice increased without treatment and decreased with AZ treatment, while levels in NSG mice decreased without treatment and increased with AZ treatment. (**D**) *Il1β* levels in WT mice slightly increased without and with treatment, while in NSG mice levels increased without treatment and decreased with AZ treatment. (**E**) *Nfkb1* levels in WT mice increased without treatment and decreased with AZ treatment, while in NSG mice levels decreased without and with treatment.

*Bax*. WT mice: The *Bax* expression levels increased without treatment (2.14 ± 1.98-fold, n = 5) and decreased with the AZ treatment (0.93 ± 0.69-fold, *p* = 0.23, n = 5). NSG mice: The *Bax* expression levels were reduced without treatment (0.766 ± 0.585-fold, n = 2) and further decreased with the AZ treatment (0.385±0.632-fold, *p* = 0.149, n = 3) (Figure 5A).

*Ho-1*. WT mice: The *Ho-1* expression levels increased without treatment (4.00 ± 3.26-fold, n = 3) and decreased with the AZ treatment (1.55 ± 1.20-fold, *p* = 0.29, n = 3). NSG mice: The *Ho-1* expression levels increased without treatment (2.66 ± 2.78-fold, n = 2) and decreased with the AZ treatment (0.412 ± 0.563-fold, *p* = 0.379, n = 2) (Figure 5B).

*Sod1*. WT mice: The *Sod1* expression levels increased without treatment (2.16 ± 2.92-fold, n = 5) and decreased with the AZ treatment (1.08 ± 0.96-fold, *p* = 0.44, n = 9). NSG mice: The *Sod1* expression levels decreased without treatment (0.256 ± 0.422-fold, n = 3) and increased with the AZ treatment (1.30 ± 1.188-fold, *p* = 0.225, n = 3) (Figure 5C).

*Il1β*. WT mice: The *Il1β* expression levels slightly increased without and with treatment, (1.1 ± 0.92-fold and 1.4 ± 0.33-fold, respectively; *p* = 0.41, n = 4, n = 5, respectively). NSG mice: The *Il1β* expression levels increased without treatment (2.236 ± 2.59-fold, n = 2) and decreased with the AZ treatment (0.986 ± 1.70-fold, *p* = 0.283, n = 3) (Figure 5D).

*Nfkb1*. WT mice: The *Nfkb1* expression levels increased without treatment (2.01 ± 1.77-fold, n = 5) and decreased with the AZ treatment (1.39 ± 1.37-fold, *p* = 0.23, n = 9). NSG mice: The *Nfkb1* expression levels decreased without and with treatment (0.372 ± 0.343-fold and 0.367 ± 0.771-fold, respectively; *p* = 0.158, n = 3, n = 5, respectively) (Figure 5E).

## 3. Discussion

AZ is a macrolide that was recently found to have potential neuroprotective effects in animal models [13,14]. On the assumption that its mode of action which is still unclear is related to immunomodulation, we sought to evaluate the effect of AZ in an ONC model of WT and immunodeficient NSG mice. Histologically and in immunohistochemistry, we demonstrated a neuroprotective effect of AZ in both groups of mice, with no effect on retinal thickness. However, there was a difference between the WT and NSG mice in molecular expression, as the AZ effect was relevant only to the WT mice while the NSG mice were not influenced by stress-related or inflammatory-related gene expression levels. In both groups, the apoptosis-related gene *Bax* was reduced in the retinas following AZ treatment, also shown by TUNEL staining. The increased *Bax* levels in the optic nerves in both the WT and NSG mice can be associated with oligodendrocyte loss directly from ONC [15].

To support the possible neuroprotective effect of AZ via immunomodulation and to investigate the underlying pathophysiological mechanisms, we studied inflammation- and stress-related genes in WT and NSG immune deficient transgenic mice. The inclusion of the transgenic NSG mice, which lack B-, T-, and NK-cells, in the analysis was intended to obtain information on the role of the immune response, which has been implicated in ONC [16]. Our results demonstrated significant differences in the molecular expression levels between the WT and immunodeficient NSG mice only in stress-related and inflammatory-related gene expression, while the *Bax* and *Ho-1* levels behaved similarly in both the retina and optic nerve. These differences, as expected, support AZ’s role as an immunomodulator. The major molecular effect was detected in the optic nerves and less so in the retina, while the microglial activation and apoptosis were clearly demonstrated in retinal immunostaining.

*Nfkb1* expression increases when the tumor necrosis factor (TNF) pathway is activated. The role of the TNF pathway in the neuroprotection of RGC survival was established by Mac Nair et al. [17]. The present study showed that treatment with AZ activated the protective effect of *Nfkb1* following the ONC-induced inflammatory reaction, only in the optic nerves of WT mice.

Previous studies of stress-related genes showed that SOD1 enzymatic activity increases during oxidative stress, such as that induced by ischemia-reperfusion injury [18] or in optic neuropathies [19,20]. In the present study, *Sod1* levels were increased in the injured optic nerves of the WT and NSG mice compared to the healthy internal control nerves and further increased in the WT AZ treated mice, but not the NSG mice, suggesting that it may reduce stress-related damage. In the retina, AZ treatment was associated with *Sod1* return to baseline in both WT and NSG mice. In the present study, the difference in the ON expression levels of *Sod1* between the WT and NSG mice may suggest a different response to injury and AZ treatment, implying differences in the response to oxidative stress between WT and NSG mice.

The levels of *Ho-1*, another stress-related gene with an attributable neuroprotective role, have also been found to increase significantly during oxidative stress in the optic nerves, in line with published data related to optic nerve damage [21]. Kutty et al. [22] reported that *Ho-1* levels were barely detectable under normal circumstances in the retina, but increased markedly in mice exposed to intense visible light compared to unexposed controls. We found in the retina increased levels (4- and 2.6-fold, WT and NSG, respectively) which returned to baseline following the AZ treatment.

Previous studies from our group reported an increase in *Ho-1* expression in the retina under extreme stress (central retinal artery occlusion) [23,24,25] and its reduction following treatment with brimonidine [25]. In an ONC model, the *Ho-1* level increased on day 3 with a hyperbaric chamber treatment [26].

Inba et al. [14] examined several macrolides, including AZ, in transient cerebral ischemia, but they administered the treatment before the induction of the ischemic damage. We injected AZ intraperitoneally immediately after the ONC induction. Its significant effect suggested that ONC leads to an immediate lymphocyte/macrophage/microglia immune reaction that can be controlled or reduced by AZ. Preventive treatment for acute optic neuropathy is possible only under laboratory conditions and not in real-life clinical scenarios. Therefore, based on our findings, we assume that the earlier the treatment is given, the greater the effect. Under laboratory conditions, the treatment administered almost concurrently with the injury revealed the maximal therapeutic effect that might be achieved. Further studies are needed to determine whether neuroprotection is achieved when the injection is given one to three days after damage. Inba et al. [14] used immunodeficient Sprague–Dawley rats, similar to NSG mice, to examine the effect of AZ and reported a limited inflammatory response.

A similar study by Varano et al. [13] investigating the effect of intraperitoneal AZ on retinal ischemia in a male Wistar rat model found that it had a neuroprotective effect. Ours is the first study, to the best of our knowledge, to examine an ONC model in NSG mice, which are usually used to investigate cancer, not ischemic or inflammatory diseases [27,28,29]. We propose that NSG mice might also serve for the study of the latter conditions, as a neuroinflammatory response is relevant in a wide spectrum of ocular diseases, including diabetic retinopathy [30].

This study revealed the role of the inflammatory system in response to damage. By including the NSG mouse groups, we were able to compare the effect of AZ on ONC-induced damage in WT mice and isolate the immunomodulating effect. The lesser effect of AZ in NSG mice, especially in the gene expression of the optic nerves, may indicate that in the presence of a compromised immune system, the inflammatory damage caused by ONC was reduced due to a decreased immune response. The effect of AZ on RGC survival and gene expression in WT mice suggests that it acts not only as an immunomodulator but also as an anti-inflammatory and anti-apoptotic agent. The possible neuroprotective effect suggested in this study may lead to novel immunomodulation treatments for optic neuropathies.

This study has several limitations. Although we clearly showed a protective effect of AZ, not all changes in gene expression were statistically significant. Additionally, there was a limited number of NSG mice, and all were female, as opposed to WT males. Furthermore, additional pathways may be involved in the neuronal and RGC damage induced by ONC.

## 4. Materials and Methods

### 4.1. Animals

A total of 68 mice were included in the study: 44 male C57/Bl6 wild-type (WT) mice (weight 24–26 g) obtained from Envigo RMS Laboratories (Jerusalem, Israel) and 24 female immunodeficient NOD scid gamma (NSG) mice from a self-colony. All mice were maintained and handled in accordance with the ARVO Statement for the Use of Animals in Ophthalmic and Vision Research and the National Institute of Health guidelines. The animal protocols for the study were approved by the local institutional animal research committee (Rabin Medical Center, RMC-020619).

### 4.2. Experimental Design

ONC was induced in the right eyes of all the animals. Animals were placed under general anesthesia by intramuscular injection of combined ketamine/xylazine (80 and 4 mg/kg, respectively) supplemented with topical proparacaine hydrochloride 0.5%. Forceps were inserted ∼2.5 to 3.0 mm posterior to the right globe, and the right optic nerve was crushed 3 times for 7 s each, separated by a 3 s interval, as previously described by our group [23]. Mice were divided into four groups: two treated with AZ (WT = 23, NSG = 12), and two untreated controls (WT = 21, NSG = 12) (Table 1). Immediately after ONC induction, mice allocated to the treated groups were given a single IP injection of AZ (Zithromax^®^, azithromycin dehydrated for injection; Pfizer, New York, NY, USA) dissolved in saline (0.9% NaCl) at a dose of 50 mg/kg, as reported elsewhere [31]. The control groups were injected with the same amount of saline only.

### 4.3. Tissue Collection

Mice were euthanized by carbon dioxide asphyxiation at 3 or 21 days after ONC/treatment, and the eyes (globes and nerves) were enucleated for molecular and histological analysis.

### 4.4. Molecular Analysis (Day 3)

Three days following ONC induction, retinas and optic nerves were dissected from both eyes (WT = 5, NSG = 5) and placed in RNAlater solution (Invitrogen, Life Technologies, Carlsbad, CA, USA) at −80 °C. Total RNA was isolated using a reagent (TRIzol; Invitrogen, CA, USA) according to the manufacturer’s protocol and then reverse-transcribed into cDNA using random hexamers (Bioline, London, UK) and Moloney murine leukemia virus (M-MLV)-reverse transcriptase (Promega, Madison, WI, USA).

Two-stage real-time quantitative polymerase chain reaction (PCR; sequence detection system, Prism 7900; Applied Biosystems, Foster City, CA, USA) was used to evaluate levels of mRNA expression of genes coding for proteins involved in apoptosis, ischemia, and oxidative stress: *Bax*, superoxide dismutase 1 (*Sod1*), heme oxygenase 1 (*Ho-1*), interleukin 1 beta (*Il1β*), and nuclear factor-kappa B 1 (*Nfkb1*). Mouse glyceraldehyde 3-phosphate dehydrogenase (*Gapdh*) was used to normalize cDNA input levels. The primers are listed in Table 2. Reactions were performed in a 20 μL volume containing 4 μL cDNA, 0.5 μM each of forward and reverse primers and buffer included in the master mix (SYBR Green I; Applied Biosystems, Foster City, CA, USA). Duplicate reactions were performed for each gene to minimize individual tube variability, and an average was taken for each time point. Threshold cycle efficiency corrections were calculated, and melting curves were obtained using cDNA for each gene PCR assay.

PCR cycling conditions consisted of an initial denaturation step of 95 °C for 10 min followed by 40 cycles of 15 s of denaturation at 95 °C and 1 min of annealing and extension at 60 °C. Standard curves were obtained using untreated mouse cDNA for each gene PCR assay. The results were quantified using a comparative threshold cycle (Ct) method, also known as the 2−ΔΔCt method, where: ΔΔCt = ΔCt (sample) − ΔCt (reference gene).

Molecular analysis was performed comparing the right (ONC) and left (control) optic nerves and retinas with and without AZ treatment for each of the WT and transgenic NSG groups.

### 4.5. Histological Analysis

#### 4.5.1. Hematoxylin and Eosin Staining (Day 21)

At 21 days after ONC induction, the eyes were enucleated and fixed in 4% formaldehyde for 1 h, washed in phosphate-buffered saline (PBS, 1X; Beit HaEmek, Israel), and placed in 15% and 20% sucrose dissolved in PBS for 1 h each. Eyes were then placed in 30% sucrose at 4 °C for 12 h and embedded in optimum cutting temperature compound (Sakura Tissue-Tek, Tokyo, Japan). Cryosections of the globes and optic nerve (6 μm) were mounted on slides and stained with hematoxylin and eosin (H&E), with three consecutive sections on each slide.

#### 4.5.2. Retinal Ganglion Cell (RGC) Count and Retinal Thickness Measurement

H&E stained slides were examined under a light microscope (Ernst Leitz GMBH Wetzlar, Germany). RGC count was determined by counting the nuclei of the RGC in the RGC layer (horizontal counting) in three sections of every 10 slides (30 consecutive sections), for a total of 7 to 10 slides per eye. The percentage of cell loss was calculated as follows: retinal cell loss = 100 * (1 − [average left-eye cell count in the RGC layer/average right-eye cell count in the RGC layer]). Retinal thickness was measured in each section by drawing a vertical line under light microscopy guidance from the outer segment of the photoreceptors, avoiding artificial detachment from the retinal pigment epithelium to the retinal nerve fiber layer internal limiting membrane.

#### 4.5.3. GFAP and CD45 Immunostaining

Cryosections of the enucleated eyes taken on day 21 were washed with PBS × 1, blocked with 2% BSA in PBS with 0.5% Triton X-100 for 15 min and incubated at 4 °C overnight with the primary antibody, rat anti-CD45 (1:100, Millipore, Temecula, CA, USA), and GFAP (1:200, Proteintech, Thermo Fisher Scientific, MA, USA). The sections were washed with 0.2% PBS with 0.5% Triton X-100 and incubated at room temperature for 1 h with the secondary antibody, goat anti-rat IgG Alexa Fluor 488 (1:200) (Molecular Probes, Invitrogen). The sections underwent nuclear counterstaining with DAPI (Invitrogen). Images were generated using a conventional fluorescence microscope (Fluoview X; Olympus, Tokyo, Japan). Excitation wavelengths were 405 nm for DAPI and 488 nm for Alexa.

#### 4.5.4. In Situ TdT-Mediated dUTP Nick End-Labeling (TUNEL) Immunostaining

Retinal cryosections 10 μm thick were cut in the direction of the optic nerve axis on days 1 and 3 following ONC with or without AZ treatment and examined by in situ TdT-mediated dUTP nick end-labeling (TUNEL) assay (Roche Diagnostics GmbH, Germany, Cat. No: 11684795910) which is a fluorescein-tagged apoptosis detection system. Staining was performed according to the manufacturer’s instructions. The sections underwent nuclear counterstaining with DAPI. Results were analyzed with a confocal fluorescence microscope (LSM 700 Inverted, Carl Zeiss, Oberkochen, Germany) equipped with appropriate filters. Excitation wavelengths used were 405 nm for DAPI and 488 nm for Cy2. The mean number of TUNEL-positive cells was determined in five different regions in the section and plotted as a column chart with standard deviation.

#### 4.5.5. IBA1 Immunostaining

Cryosections of the enucleated eyes taken on day 3 were washed with PBS × 1, permeabilized with 1% Triton for 10 min, blocked with 5% Fetal calf serum in PBS for 60 min, and incubated at 4 °C overnight with the primary antibody, rabbit anti-IBA1 (1:500, Abcam, Cat# ab178846). The sections were washed with PBS ×1 and incubated at room temperature for 1 h with the secondary antibody, goat anti-rabbit IgG H&L Alexa Fluor 647 (1:1000, Abcam, Cat# ab150079). The sections underwent nuclear counterstaining with DAPI (Sigma Aldrich Israel, Rehovot, Israel). Images were generated using an LSM 700 inverted confocal fluorescence microscope (Carl Zeiss, Oberkochen, Germany). Excitation wavelengths used were 405 nm for DAPI and 647 nm for Alexa Fluor.

### 4.6. Statistical Analysis

Differences between groups were analyzed using an unpaired Student’s *t*-test. Significance was defined as *p* < 0.05.

## 5. Conclusions

This study demonstrated important differences between WT mice with a normal immune response and immunodeficient NSG mice subjected to ONC. AZ had a neuroprotective effect against ONC-induced damage that preserved the RGCs in the WT mice. This effect was not significant in the NSG mice, which had a lower (baseline) level of inflammatory markers following ONC. AZ treatment reduced the expression of stress-related genes and modulated the inflammatory reaction in the retina while increasing it in the optic nerves. This macrolide drug, already available and FDA-approved for infections, may potentially protect oxidative-stress-related acute optic neuropathies. We suggest that the neuroprotective effect is instigated by immunomodulation, as indicated by the improved response of the WT mice to the AZ treatment compared to the NSG mice.

## Figures and Tables

**Table 1:** Experimental design.

Procedure/Study	Analysis Method	C57Bl/6 Mice (n = 24)	NSG Mice (n = 24)
ONC	molecular	10	5
	IHC	11	7
ONC + AZ	molecular	10	5
	IHC	13	7

ONC = optic nerve crush, AZ = azithromycin, IHC = immunohistochemistry; NSG = NOD scid gamma.

**Table 2:** List of primers for molecular studies.

*Sod1*_F	GCCCGGCGGATGAAGA
*Sod1*_R	CGTCCTTTCCAGCAGTCACA
*Bax*_F	CTGAGCTGACCTTGGAGC
*Bax*_R	GACTCCAGCCACAAAGATG
*Ho-1*_F	CAGGTGTCCAGAGAAGGCT
*Ho-1*_R	TCTTCCAGGGCCGTGTAGAT
*Il1β* _F	TGACAGTGATGAGAATGACCTGTTC
*Il1β* _R	GGACAGCCCAGGTCAAAGG
*Gapdh*_F	TGCCACTCAGAAGACTGTGGATG
*Gapdh*_R	GCCTGCTTCACCACCTTCTTGAT
*Nfkb1*_F	CCTGCAAAGGTTATCGTTCAGTT
*Nfkb1*_R	GCAAAGCCAACCACCATGT

## Data Availability

The data presented in this study are available on request from the corresponding author.
